# Bioengineering advancements, innovations and challenges on green synthesis of 2, 5-furan dicarboxylic acid

**DOI:** 10.1080/21655979.2019.1700093

**Published:** 2019-12-27

**Authors:** Rajendran Omana Rajesh, Tharangattumana Krishnan Godan, Raveendran Sindhu, Ashok Pandey, Parameswaran Binod

**Affiliations:** a Microbial Processes and Technology Division, CSIR-National Institute for Interdisciplinary Science and Technology (CSIR-NIIST), Thiruvananthapuram, India; b Academy of Scientific and Innovative Research (AcSIR), CSIR-National Institute for Interdisciplinary Science and Technology (NIIST), Thiruvananthapuram, India; c Centre for Innovation and Translational Research, CSIR-Indian Institute of Toxicology Research (CSIR-IITR), Lucknow, India

**Keywords:** Green synthesis, FDCA, HMF, biological, enzymes, lignocellulosic biomass, renewable resources

## Abstract

The major drawback of chemical transformations for the production of 2, 5-furan dicarboxylic acid (FDCA) implies the usage of hazardous chemicals, high temperature and high pressure from nonrenewable resources. Alternate to chemical methods, biological methods are promising. Microbial FDCA production is improved through engineering approaches of media conditions, homologous and heterologous expression of genes, genetic and metabolic engineering, etc. The highest FDCA production of 41.29 g/L is observed by an engineered *Raultella ornitholytica* BF 60 from 35 g/L HMF in sodium phosphate buffer with a 95.14% yield in 72 h. Also, an enzyme cascade system of recombinant and wild enzymes like periplasmic aldehyde oxidase ABC, galactose oxidase M3-5, HRP and catalase have transformed 6.3 g/L HMF to 7.81 g/L FDCA in phosphate buffer with 100% yield in 6 h. Still, these processes are emerging for fulfilling the industrial needs due to the challenges in ‘green FDCA production’.

## Graphical Abstract

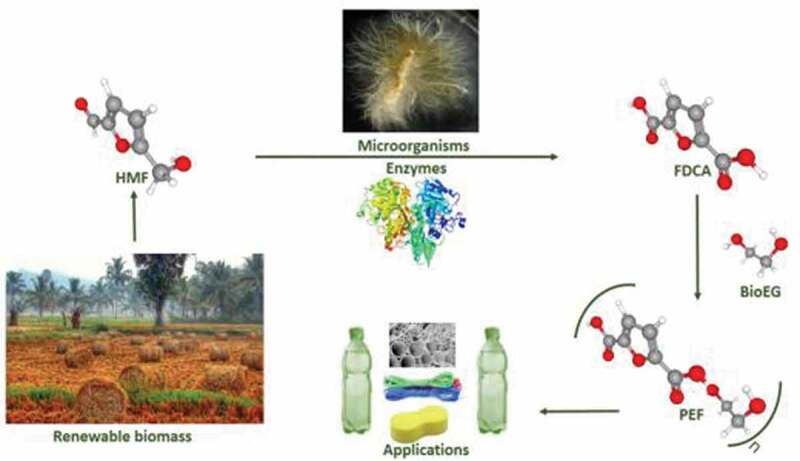

## 1. Introduction

Lignocellulosic biomass is a potentially renewable resource and can be utilized to synthesize green chemicals based on biological approaches [1]. Also, the U.S. Department of Energy and the U.S. Department of Agriculture have a vision of a 30% reduction in U.S. petroleum consumption with biofuels by 2030 [2]. Biomass from agricultural resources can be used as one of the best renewable energy sources in the world instead of chemicals and fuels from nonrenewable sources. As a feedstock, lignocellulosic biomass is used for the production of plastics, paper, pulp, fuels and chemicals in biorefineries which will eventually boost up the rural economy and farmers also. This will reorient the direction of chemical industries from petroleum refinery to lignocellulosic biomass refinery and lead to the competitive production of bio-based products [3]. Major unused biomass is in the form of crop waste, forest residues, paper waste etc [4,5]. This biomass is mainly composed of cellulose, hemicellulose, lignin, and other constituents [6]. Among them, cellulose is insoluble in water and made up of glucose subunits by β-1,4 glycosidic bonds [7,8]. Hemicelluloses are composed of monomers of xylose, arabinose, mannose, glucose, etc [9,10]. The depolymerization of cellulose and hemicellulose by acid or alkali pretreatment, produce glucose which can be transformed into many value-added chemical products. This glucose is also used by biorefineries for the production of biochemicals, biopolymers, and biofuels. One of the main platform chemicals transformed from glucose or fructose is Hydroxymethyl furfuraldehyde (HMF) [11,12].

**Figure 1.**
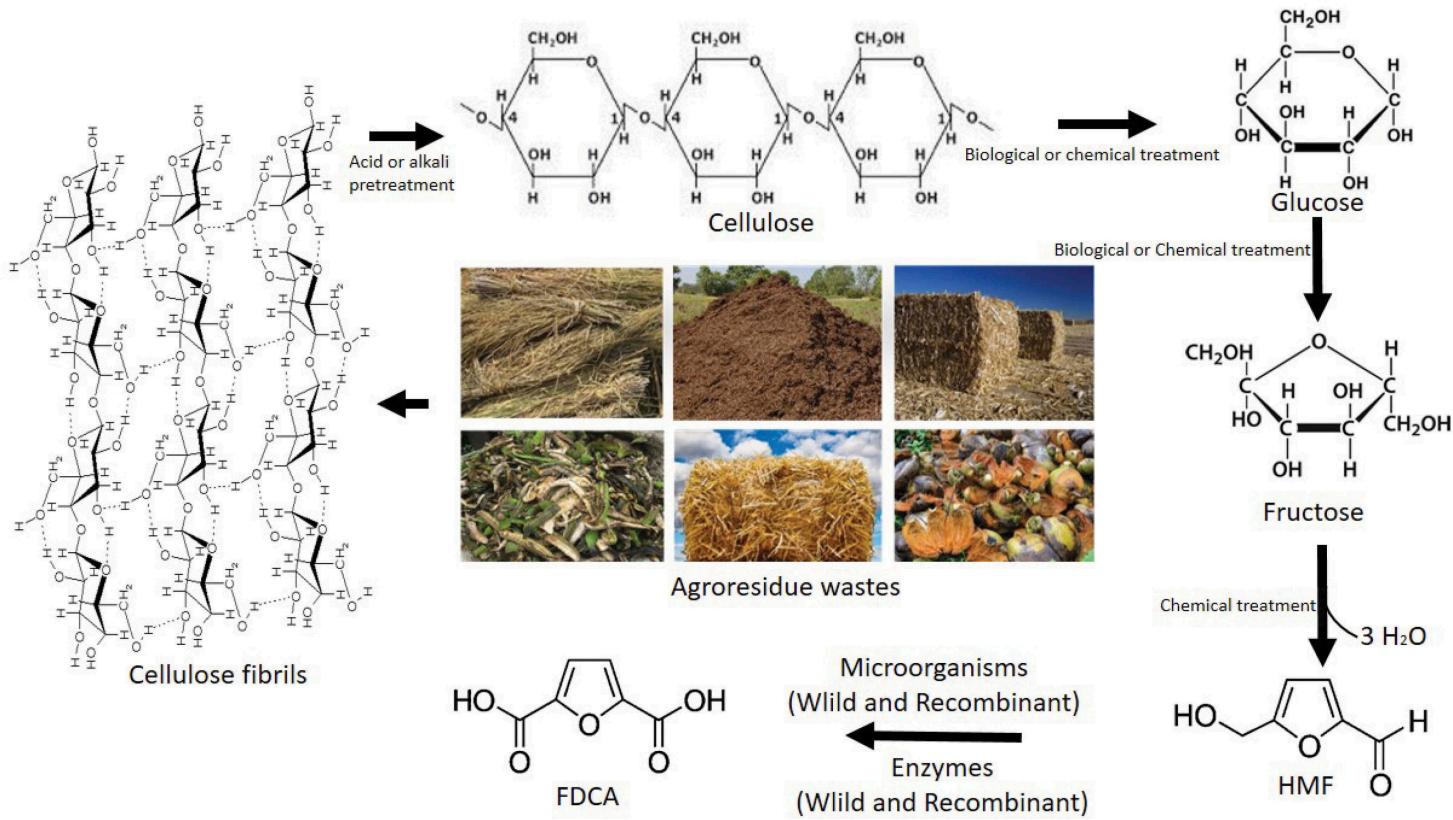
Conversion of agro-residue wastes or biomass into HMF through chemical catalysis followed by FDCA production through biological processes.

Even though fructose conversion to HMF using chemical strategies is simple process when compared to glucose or lignocellulosic biomass, the latter can be used as sound substrates. Polysaccharides such as starch [13], cellulose [14], chitin [15], and inulin [15] have also been used as potential feedstocks for the HMF production using chemical catalysts. All these feed stocks requires a common step of depolymerization of glucose through hydrolysis followed by dehydration leading to the formation of HMF [16] (Figure 1). The depolymerized glucose from biomass or the natural free glucose is isomerized into fructose using chemical catalysts like titanium dioxide (TiO_2_) and zirconium oxide (ZrO_2_) under microwave irradiation [17]. It has been reported that the isomerization of glucose to fructose is done using immobilized glucose isomerase [18]. Reports claim that HMF production from these substrates has been done using solid catalysts like aluminum sulfate and aluminum chloride, sulfate/zirconium oxide – aluminum oxide, zirconium phosphate, zirconia, aluminum chloride, ion-exchange resins, stannic chloride, Sn-Mont [13,19,20], lanthanide, niobium and organic-inorganic nanocomposite based catalysts etc [21–25]. Due to the specialties like the easiness of separation, recyclability, reaction fastness, sustainability (even at higher temperatures) and enhanced selectivity these solid catalysts are industrially preferable [19,26]. HMF could also be synthesized from raw biomass, glucose and fructose using ionic liquids like1-octyl-3-methylimidazolium ([OMIM], 1-hexyl-3-methylimidazolium ([HMIM]), 1-butyl-3-methylimidazolium ([BMIM]), 1-ethyl-3-methylimidazolium ([EMIM]) etc [27,28]. Ionic liquids have advantages like high stability, low vapor pressure and recyclability [27,28]. The yields of HMF from monosaccharides or polysaccharides or biomass are comparatively lower than those obhtained from fructose. Natural processes like caramelization [29] and Maillard reactions [30,31]form HMF as an intermediate.

HMF is a versatile platform chemical for the manufacture of 5-hydroxymethylfuroic acid, bishydroxymethylfuran, HMF diethers, 2, 5-dimethylfuran, alkoxymethylfurfuran and 2, 5-furandicarboxylic acid (FDCA). These furan derivatives have valuable fuel and/or polymer applications. Also, adipic acid, 1, 6-hexanediol, levulinic acid, caprolactam and caprolactone are the non-fumaric compounds produced from HMF [32]. Among them, FDCA is the most industry liked chemical with a broad range of applications compared to other HMF derived products. The similarity of its aromatic counterparts makes it as a different structure feature from other chemicals for the polymerization applications [33]. Rudolph Fittig and Heinzelmann in 1876 reported FDCA as dehydromucic acid, who produced it by the action of concentrated hydrobromic acid and mucic acid [34]. However, the progress research about FDCA synthesis and its application studies were discontinued and were only resumed in the last 10 years. With the emergence of the biorefinery concept, there was a massive move from nonrenewable fossil-based processes to sustainable renewable resource processes. According to a study conducted by USA National Renewable Energy, the conversion of renewable biomass to platform chemicals has been reported. One of the key green or bio-based platform chemical in that list is, FDCA [35]. It has been considered and called as a ‘sleeping giant’ due to its applications in diverse areas [26,36].

The present review discusses recent developments in the green synthesis of FDCA through biological methods. This biological biotransformation of HMF into FDCA is done mainly through microorganisms or enzymes. The mechanism of biotransformation of HMF to FDCA in prokaryotes and eukaryotes is elucidated here which will pave the way for full mechanism in the coming years. Microbial FDCA production is done either by direct use with media engineering approaches, recombinant microorganisms, metabolic pathway engineering and engineering of membrane-bound transport proteins for the transport of pathway intermediates, etc. Currently, this biotransformation has been progressive through processes using recombinant enzymes and wild enzymes also. Even though the use of microorganisms and enzymes make the processes greener, these processes are not economically viable with chemical transformations of HMF to FDCA for the large scale production.

## 2. Applications of FDCA

HMF is metabolized into FDCA in human urine and blood plasma in a negligible amount. Major applications have been summarized in Table 1. Mainly FDCA can be used for the production of biochemical like succinic acid [37], isodecylfuran-2, 5-dicarboxylate [38], isononyl furan-2, 5-dicarboxylate [39], dipentyl furan-2, 5-dicarboxylate, diheptyl furan-2, 5-dicarboxylate and poly (ethylene dodecanedioate-2, 5-furandicarboxylate) (PEDF) [40]. FDCA is also an important ingredient in the preparation of hexanoic acid, macrocyclic ligands [41], fungicides [38], corrosion inhibitors and thiolene films [42,43]. FDCA derivatives like 2, 5-dihydroxymethylfuran 2, 5-bis (hydroxymethyl) tetrahydrofuran could be used in the production of new polyesters as alcohol components. It is a highly demanding monomer in the production of dichloride- (FDCDCl), dimethyl- (DMFDC) and bis (hydroxyethyl)- (BHEFDC) derivatives for the production of plasticizers, polyamides, and polyesters [44]. Structurally furan rings of FDCA are analogous to fossil derived TPA (Terepthalic Acid) [45] which is one of the currently used plastic material widely used today and FDCA can be an eco-friendly alternative for the production of new bioplastics [46]. It can also be used as an alternative for polybutylene terephthalate (PBT) and polyethylene terephthalate (PET) [47] which are utilized in the production of film, fiber, packing materials and soft drink bottles [48,49]. FDCA monomer as such cannot use in polymer production. This has to be combined with ethylene glycol and synthesize PEF (polyethylene furonate) and PBF (polybutylene furonate). Due to the thermo-chemical, mechanical, gas barrier and recyclability properties of PEF this can be used as an alternative of PET and PBT [50].

**Table 1.** Applications of FDCA.

Sl.No	Chemicals made of FDCA	Applications of FDCA based chemicals
1	Succinic acid, Isodecylfuran-2, 5-Dicarboxylate, Isononyl furan-2, 5-dicarboxylate, Dipentyl furan-2, 5 dicarboxylate, Diheptyl furan-2, 5-dicarboxylate and PEDF	Biochemicals
2	Hexanoic acid, Macrocyclic ligands, Fungicides, Corrosion inhibitors and Thiolene films	Ingredients
3	2, 5-Dihydroxymethylfuran 2, 5-Bis (hydroxymethyl) tetrahydrofuran	Polysters
4	Dichloride- (FDCDCl), Dimethyl- (DMFDC) and Bis (hydroxyethyl)- (BHEFDC)	Monomers of Plasticizers, Polyamides and Polyesters
5	PEF (polyethylene furonate) and PBF (polybutylene furonate)	Film, Fiber, Packing materials and Soft drink bottles

There was not much production of PEF from industries due to its less purity, sustainable availability and the process difficulties of FDCA. FDCA diethyl esters have anesthetic properties similar to cocaine. It has a chelating property with ions (Ca^2+^, Cu^2+^ and Pb^2+^) and applied as a medicine for the kidney stones removal [51], synthesize of artificial vein transplantation. Ultimately this FDCA has been highlighted as one of the 12 platform biochemical used for the in-depth research for sustainable industrial production.

## 3. Industries involved in FDCA production

The industrial FDCA production is started by Avantium using the ‘YXY’ process which includes carbohydrate dehydration to alkoxymethylfurfural (RMF) or methoxymethylfurfural (MMF), followed by oxidation to FDCA. This FDCA and ethylene glycol combined in the last step for the production of PEF. Their pilot plant production started in 2011 aiming with a target of 40 tons of FDCA per year. After this success, Avantium has established a joint consortium with BASF (Synvina) and a pilot plant designed for FDCA production with a capacity of 50,000 tons per year with PEF production. Apart from that, the company Corbion has used a microbial process for large scale FDCA production from HMF. A joint consortium of Corbion and BASF called Succinity has efficiently replaced by using succinic acid, which is a petrochemical product. DuPont joint venture with Archer Daniels Midland Company (ADM) has developed a process to produce FDCA esters in 2016. HMF has been oxidized at high pressure at 115°C. In 2017, a commercial process for FDCA production is developed by Origin Materials and Eastman Company.AVA Biochem’s FDCA production capacity of approximately 30,000 tons per year has started in 2019 and this aim will then be increased to 1,20,000 tons per year. Petrobras has been developed a two-step process in which the first step of HMF production is from sucrose, glucose and fructose (C6 sugars) followed by FDCA conversion using resin as the second step. VTT Technical Research Center has developed a biological industrial production from hexaric acid using modified uronate dehydrogenase enzyme for the conversion of the substrate D-galacturonic acid into mesogalactaric acid (mucic acid) and yet to produce a high amount of FDCA [52]. So globally the FDCA and its derivatives together contributes a commercial estimated production of 4 metric tons per year which may cost approximately a market value of 40 million USD from estimated FDCA production of 9 billion lb/year. This will give a value of $0.85 to 2.20/lb [35]. This global market will reach to 498.15 kilotons of FDCA production in the coming years.

The majority of the industries depend on chemical processes for the production of FDCA from different substrates. Each technology has its drawbacks and limitation affecting its successive large scale production. Among the above industries, only BASF got 95.2 % of FDCA yield from HMF in D_2_O using Pt/C catalyst at 100 bar air pressure at 100°C. All other industrial chemical technologies have used expensive catalysts, high temperature and high pressure. These processes usually carried out in D_2_O or other solvents like methanol or acetic acid using HMF as the substrate with less FDCA yield. Their conditions, expensive substrates, expensive or toxic catalysts make the processes non eco-friendly and economically non viable with less product yield. Corbion and VTT (research center) have used greener technologies so far. Corbion use microbial biotransformation technology to produce HMF from FDCA. VTT developed a partial biological technology with very little FDCA yield (1.7 %). VTT has produced FDCA from galataric acid with methyl trioxorhenium catalyst in methanol with less pressure (1 bar) at high temperature (100°C). So the main problems like environment toxicity using chemical catalysts, chemical media and hazardous conditions have been overcome through biological technologies. Still, the existing biological technologies also have their demerits like less yield and longer time.

## 4. Biotransformation mechanism of HMF into FDCA in microorganisms

**Figure 2.**
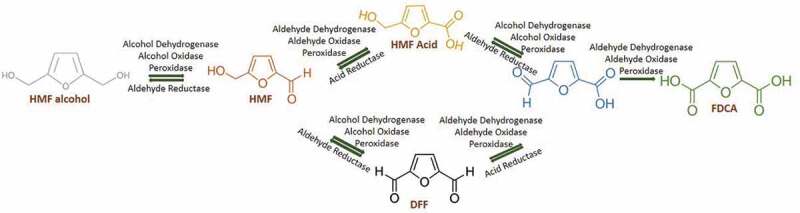
Green synthesis of HMF into FDCA through enzymes.

Fructose dehydrated HMF molecule contains a furan ring with both aldehyde and alcohol functional group on both sides. This aldehyde or alcohol functional group makes this a toxic molecule to living organisms. Furanic aldehydes in this respect causing ROS-associated damage to nucleic acids, proteins and cell organelles of microorganisms [53,54]. But some microorganisms like bacteria, fungus (white rot and mycelial) and algae have natural detoxification mechanisms generally to xenobiotics, aldehydes and alcohols. Still, degradation or detoxification of HMF is naturally present for some microorganisms only. HMF detoxification is present in bacteria and fungus mainly can be exploited for industrial applications. Degradation of HMF proceeds through FDCA production (Figure 2) which is an industrial relevant compound with many applications. So microorganisms like bacteria and fungus are used for FDCA production from HMF. Even though the actual mechanisms of this are still unknown, there are reports for its mechanism in prokaryotes and eukaryotes are illustrated here with available information.

### 4.1. Biotransformation of HMF in prokaryotes

**Figure 3.**
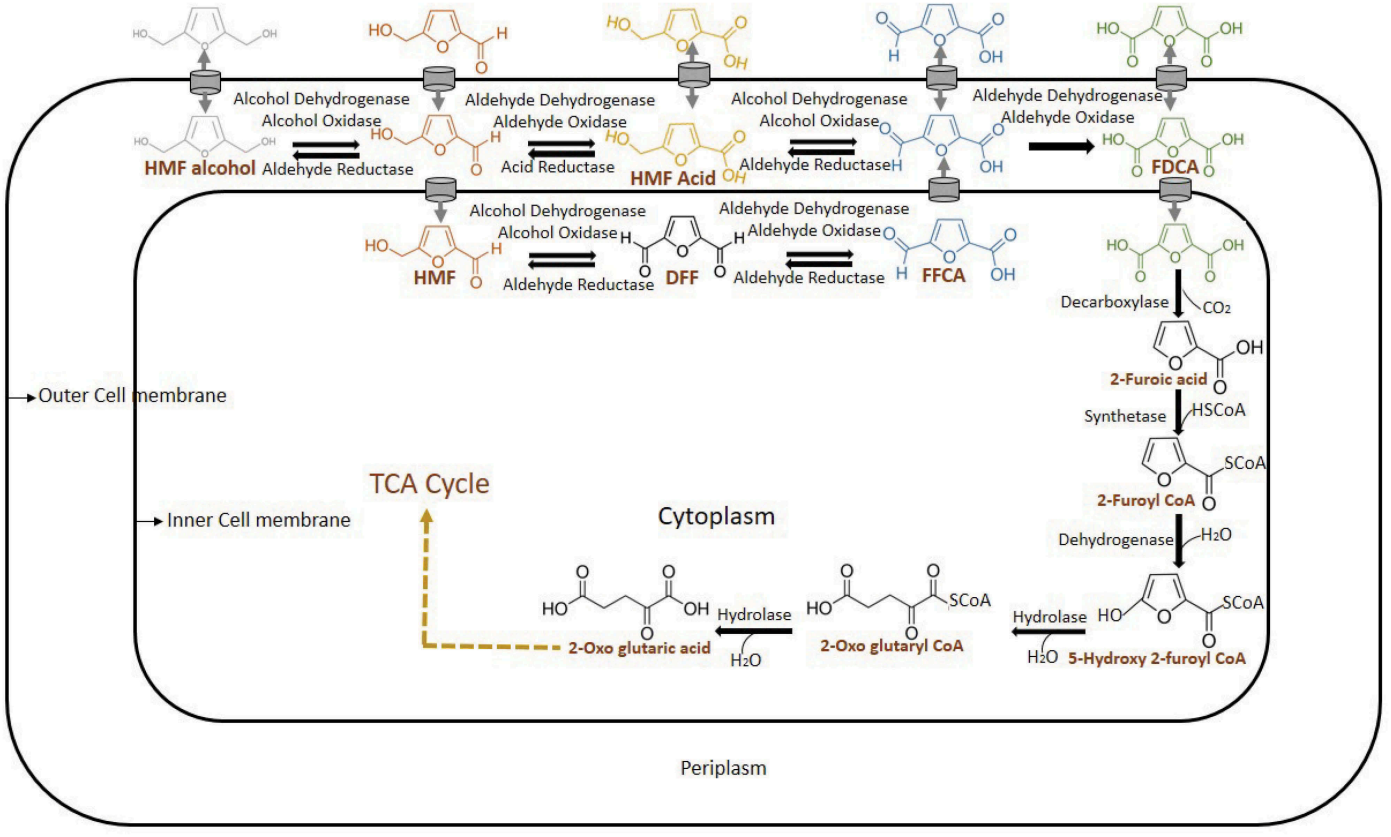
Mechanism of biotransformation of HMF into FDCA in bacteria.

HMF biotransformation or detoxification through FDCA is firstly reported in *Cupravidus basilensis* HMF 14. In this organism, this process occurs through HMF degradation gene clusters *hmfABCDE* and *hmfFGH’H*. If the hmfABCDE gene cluster is mutated, the growth of organisms not occurred in either HMF or furfural carbon source, suggesting a shared metabolic pathway between these two aldehydes. If *hmfFGH’H* gene cluster is mutated growth of *C. basilensis* HMF 14 has not occurred in HMF medium. It is concluded that the later gene cluster is only associated with HMF degradation pathway. Among this gene cluster, biotransformation of HMF to FDCA is associated with the gene *hmfH* followed by FDCA decarboxylation with *hmfG*. The genes *hmfH* and gene *hmfG* are responsible for the enzymes oxidoreductase and decarboxylase respectively [55]. From the available reports in bacteria, it can be concluded that HMF is converted into either HMF alcohol or HMF acid by reductase or oxidase respectively. If it is oxidized to form HMF acid by dehydrogenase or oxidase or peroxidase, it will proceed into the FDCA production pathway, which occurs in the periplasm (Figure 3). The reduced form of HMF (HMF alcohol) can be converted back to HMF again by dehydrogenase or oxidase or peroxidase. HMF to FDCA conversion is taken place commonly by any of these classes of enzymes (Figure 2). HMF is getting converted into HMF acid (periplasm) or DFF (intracellular) and these two converted into FFCA. Until the FFCA conversion step, all the reactions are reversible. This FFCA is getting converted into FDCA, which is non-reversible. This FDCA is up taken by the cells for its metabolic activities after decarboxylation into 2-furoic acid. The intracellular 2-furoic acid is getting converted into 5-hydroxy-2-furoyl Co A, 2-Oxoglutaroyl Co A, 2-Oxoglutaric acid and entered into TCA cycle [56]. In bacteria, usually, dehydrogenases, oxidases and peroxidases are seen in periplasmic space for the detoxification of toxic aldehydes and alcohols. Microorganisms reduce or oxidize these compounds into less or nontoxic forms for their survival and stay in the lag phase. After the biotransformation stage or FDCA production, it is entered into the metabolic pathway for its further metabolic activities.

### 4.2. Biotransformation of HMF in eukaryotes

**Figure 4.**
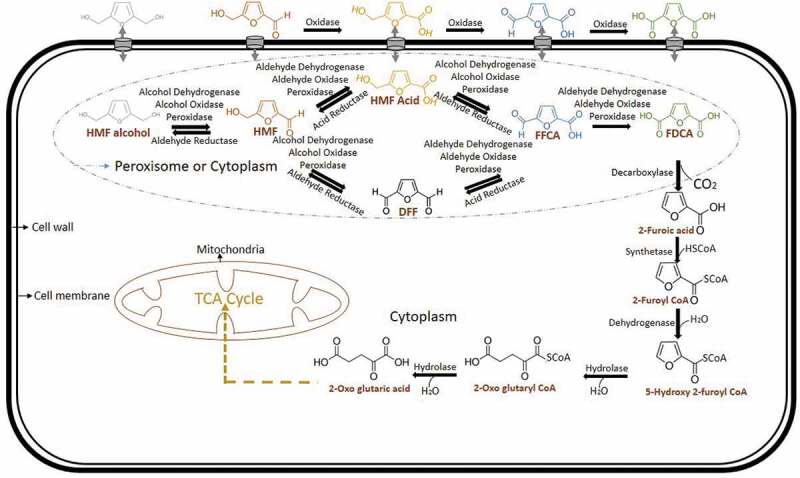
Mechanism of biotransformation of HMF into FDCA in fungi.

HMF biotransformation in fungus has been reported in basidiomycete *Pleurotus ostreatus* (white rot). This can detoxify HMF into HMF alcohol and the FDCA production pathway. It has been reported that aryl alcohol oxidase and aryl alcohol dehydrogenase are involved in this intracellular coupled with extracellular HMF biotransformation. An elevated level of aryl alcohol oxidase and aryl alcohol dehydrogenase expression is seen after HMF induction in quantitative RT-PCR. This expression profile from RNA extract is shown that these enzymes are from the genes *aao 1–3* and *aad 1* respectively. Aryl alcohol oxidase, *aao* 4 expressions are increased after 24 h of HMF induction which showed that intermediates of HMF biotransformation into FDCA [57]. Since this report says it as a reaction with intracellular coupled with extracellular HMF detoxification, recent reports are also taken into consideration for this biotransformation in which, HMF alcohol, HMF acid, FFCA and FDCA are produced extracellular after HMF intake (Figure 4). DFF and 2-furoic acid is not formed in the extracellular medium and observed in the intracellular mycelial fraction [58]. So from these reports, it has been concluded that dehydrogenases, oxidases and peroxidases of the fungal HMF biotransformation are done intracellularly also. Then, the extracellular FDCA can be up taken to fungal cells followed by 2-furoic acid pathway like bacteria.

## 5. Technological advancements and innovations for the green synthesis of FDCA

### 5.1. Engineering of media components and conditions in microbial bioprocesses for the production of FDCA

*Burkholderia cepacia* H-2 has been isolated from the enrichment isolation technique from the soil in 0.2 to 1 g/L HMF. This microorganism is capable of transforming HMF into FDCA after preliminary analysis in 10 mL mineral salt media with 0.2 g/L HMF. After media optimizations of substrate concentration ranging from 0.5 g/L to 3 g/L and transformed 2 g/L HMF to 1.2 g/L FDCA in 125 mL batch reactor. Media pH is optimized ranging from 5.0 to 8.0 and optimized pH is noted as 7.0 and 1.2 g/L HMF produced from 2 g/L HMF after 32 h. The temperature of media is optimized ranging from 26°C to 32°C and maximum FDCA of 1.27 g/L obtained at 28°C. These single parameter media optimizations improved the FDCA production up to 1.27 g/L from 2 g/L HMF after 24 h. Thermal algal acid hydrolyzate of *Chaetomorpha linum* contains HMF is transformed into FDCA by *B. cepacia* H-2. Hydrolysis of 3 % algal biomass was done using 0.5 M HCl under 121°C for 15 min for the HMF preparation. HMF (0.2 g/L), reducing sugars, furfural (30 mg/L) and acetic acid (104 mg/L) are analyzed in thermal algal hydrolyzate and this cheap medium is used for the production of FDCA. Acid algal hydrolyzate of 0, 2 and 4 times dilution are inoculated with *B. cepacia* H-2 (0.1 O.D) in batch reactor and 2 g/L HMF added into it. After incubation 1.03 g/L of FDCA obtained from two dilutions after 18 h [59]. This single parameter optimizations and the use of cheap thermal algal hydrolyzate improved the production of FDCA from HMF.

Biotransformation of HMF into FDCA is performed using the isolated strain *Methylobacterium radiotolerans* G-2. This isolated strain is enriched in 0.2 g/L HMF for the HMF utilization rates. After HPLC analysis it is confirmed that this strain could produce FDCA. Media engineering adopting single parameter optimization was carried out for enhancing the production of FDCA by *M. radiotolerans* G-2. Parameters like substrate (HMF) concentration, pH and temperature are done for increasing the FDCA production. The organism is inoculated in 40 mL of mineral salt media with a cell concentration of 0.1–0.02 O.D in 125 mL batch reactors at 120 rpm. Media optimization of substrate concentration is done ranging from 0.5 g/L to 3 g/L and best FDCA production of 1.6 g/L obtained from 3 g/L HMF after 72 h. Optimization of media pH was done ranging from 5.0 to 8.0 and a maximum of 0.5 g/L FDCA produced from pH 7.0 and 8.0 which is neutral and slightly alkaline respectively. The temperature of media is obtained ranging from 26°C to 32°C. The specific growth rate of *M. radiotolerans* G-2 is seen as 0.02–0.03 L/h in every media incubation temperature and FDCA concentration at each incubated temperature as 0.513 g/L. Since the FDCA degrading rate is lowest at a temperature of 26°C, this temperature was used throughout the studies. As part of FDCA production from renewable resources, synthetic media like mineral salt media are avoided and algal acid hydrolyzate used. This algal hydrolyzate is made from the acid treatment of unused or waste algae which contains reducing sugars, HMF, acetic acid and furfural. The organism is inoculated into 0, 2 and 4 fold dilutions. Maximum FDCA of 0.45 g/L is produced from 2 and 4 fold dilutions of thermal algal acid hydrolyzate[60]. Algal hydrolyzate has avoided the use of synthetic media and FDCA production has been improved after single parameter optimizations.

*R. ornithinolytica* BF60 is isolated from the soil enrichment isolation technique showed HMF conversion and FDCA production upon preliminary analysis. Reaction conditions are optimized for the whole-cell biotransformation of HMF into FDCA in phosphate buffer. First, the optimal pH of the whole-cell biocatalyst is determined to range from 6.0 to 10.0 and better pH found as 8.0 (slightly alkaline). The optimum temperature of this biocatalyst is done ranging from 20°C to 45°C and its maximum activity found at 30°C. Optimal substrate concentration (HMF) is done ranging from 25 to 150 mM. The maximum production of FDCA is reached (12.08 μmol L^−1^h^−1^) at 100 mM HMF. It is indicated that the activity of microbial biocatalyst gets inhibited higher than 100 mM HMF. The inoculation age of the whole-cell biocatalyst is also optimized and better FDCA production observed at the late exponential phase. At this stage, the toxic effect of HMF is minimized and conversion of HMF into the desired product FDCA is maximized. Inoculum size of the biocatalyst is determined ranging from 15 to 75 g/L and the highest FDCA production resulted in 45 g/L. At this inoculum size, the FDCA production rate maintained at 12.08 μmolL^−1^h^−1^ (9.2 g/L) from 100 mM HMF. These biochemical characters again optimized employing a fractional factorial design and similar results obtained. The study revealed that a high cell concentration does not enhance FDCA production due to the deficiency of oxygen in the media. This is because the pathway generates reducing power for the energy metabolism of the microorganisms which must be oxidized to sustain the flux [61]. This media engineering process is optimized for FDCA production by single parameter optimization and fractional factorial design, and production has been increased from preliminary results.

*Enterobacter* sp. has been isolated from acid pretreatment liquor drainage site after enrichment culture isolation in mineral salt media and HMF. The organism is grown for 48 h in nutrient broth and after that 20% (v/v) of its cell biomass is collected. This biomass is transferred into mineral salt media and HMF for the biotransformation. To optimize the media ingredients for maximum HMF biotransformation, 0.25% glucose and 0.25% glycerol is added to this media and the production rate of FDCA got delayed. Among them, media with 0.25% glucose showed much delay in the FDCA production as compared to the media with 0.25% glycerol. This might be due to the uptake of glucose into the microorganism for its metabolic activities rather than HMF biotransformation. Also, cell density is higher in the glucose enriched media with HMF as compared to a media without carbon sources. So a mineral salt media with 0.5 g/L HMF got converted into 0.07 g/L FDCA after 14 days [62]. This result shows that less substrate concentration is used with less production in 14 days after media optimizations.

*Acinetobacter oleivirans* S27, an isolate from high altitude soil, Sikkim, India was used for the biotransformation of HMF into FDCA. The strain was grown in LB medium and cell biomass collected after centrifugation. This biomass was transferred into mineral salt media with 0.5 g/L HMF. Optimization of media temperature (30°C – 37°C), pH (6.0 – 8.5), substrate concentration (0.5 – 3.0 g/L) and inoculum age (24 −28 h) are performed and observed as 30°C, 6.5, 1.5 g/L and after 27 h respectively. After a single parameter optimization 0.23 g/L FDCA obtained from 1.5 g/L HMF. Statistical design experiments adopting RSM design with three parameters were carried out. The parameters selected were inoculum age, inoculum size and pH of the media are optimized for the FDCA production and optimized as 24 h, 5.0 and 5.5 respectively. So the optimized media with these conditions produced a 65% yield of FDCA from 0.5 g/L HMF [63]. This is mainly focused on the biotransformation of FDCA and less amount of FDCA produced with less substrate detoxification.

*Aspergillus flavus* APLS-1 has been isolated from acid pretreatment liquor of the pilot plant, India and showed HMF conversion into FDCA after preliminary analysis. Screening was done in mineral salt media and 0.5 g/L HMF. The addition of other carbon sources (glucose and potato dextrose broth) other than HMF into mineral salt media and HMF showed an inhibition in FDCA production due to the uptake of normal carbon sources for its metabolic activities. About 3.0 g/L HMF is tolerated by *A. flavus* in solid agar plates without many morphological changes. Media engineering using single parameter optimization strategy was adopted to enhance the FDCA production. Media is optimized with different pH (6.0 to 8.0), cell biomass size (4.0 – 8.7 g/L), biomass age (48 – 84 h) and substrate concentration (0.25 – 2.0 g/L). Pre-grown cell biomass was added into mineral salt media containing HMF and this toxic HMF is bio-transformed into FDCA upon sequential oxidations. After this optimization maximum FDCA 0.32 ± 0.06 g/L is produced at pH 6.5, biomass size 6.0 g/L, biomass age 60 h and HMF 1.0 g/L after 14 days. After this to improve the FDCA production, a Box-Behnken design is made to know the Response surface methodology of three major parameters biomass size, biomass age and pH of the medium. Here substrate concentration (HMF) is kept as constant as 1.0 g/L. Media optimization using Box-Behnken design has improved 67% conversion efficiency of 1.0 g/L HMF (8 mM) and 0.8 g/L (6.4 mM) of FDCA is produced in 14 days with biomass size of 5.7 g/L at pH 6.5 and biomass age 60 h. It is validated after validation experiments with biomass size 6.6 ± 0.1 g/L, pH 6.5, and biomass age 60 h. This condition is validated and FDCA production of 0.83 g/L (6.6 mM) obtained from 1.0 g/L (8 mM) HMF with a yield of 67% after 14 days [58]. Even though media optimizations have been improved biotransformation of HMF into FDCA, its production occurred after 14 days.

### 5.2. Homologous and heterologous expression of genes in microorganisms by genetic engineering for the production of FDCA

#### 5.2.1. Homologous expression of genes in microorganisms

The genes in the pathway of *Raoultella ornithinolytica* BF60 for biocatalytic oxidation of HMF to FDCA has been identified by transcriptomic analysis. This demonstrated that HMF conversion to the undesired product 2, 5-bis (hydroxymethyl) furan (HMF alcohol) is associated with the genes *adhP3* and *alkR* and the desired product FDCA with *aldH*. Combinatorial deletion of two genes *adhP3* and *alkR* by λ Red recombination system resulted in an 85.7% reduction in HMF alcohol and an overall increase in FDCA (23.7%) production (242.0 mM). Amplified *aldH* fragment (20 bp flanking homologous sequences at *Nde* I and *Xho* I) has been ligated to a linearized plasmid pACYC-hmfH through recombination using the ClonExpress II Kit yielded the plasmid pACYC-*hmfH-aldH*. Biotransformation experiments have been conducted in 10 mL of pre-grown cell suspension (O.D_600_ 100) and 0.5 g Calcium carbonate (Neutralizing agent) in 50 mM sodium phosphate buffer (pH 8.0) at 30°C. Fed-batch approach of 50, 50, 50, 50, 25, 25, and 25 mM HMF are fed at every 12 h until 72 h. Over-expression of *aldH* gene responsible for the oxidation of the intermediate 5-formyl-2-furan carboxylic acid (FFCA) to FDCA has resulted in a 96.2 % yield of FDCA (264.7 mM) [64]. Even though gene expression and fed-batch approach have improved FDCA production the reaction is carried out in low reaction volume which is suitable for laboratory scale work only.

#### 5.2.2. Heterologous expression of genes in microorganisms

Gene (*hmfH*) of enzyme oxidoreductase from *Cupriavidus basilensis* HMF14 has been cloned into pJTmcs with a constitutive *tac* promoter as transcription control. The resultant plasmid pJThmfH is introduced into *Pseudomonas putida* S12 and this engineered whole-cell biocatalyst produced FDCA from HMF in the preliminary analysis itself. HMF concentration in the media increased up to 50 mM resulted in *q*FDCA from 116 ± 1.82 to 276 ± 89 µmol (g CDW)^−1^ h^−1^). HMF concentration of 75 mM or higher an intermediate HMF acid is no longer completely converted to FDCA. This may be due to the toxicity of HMF to the host strain. Moreover, the high concentrations of acid intermediates like HMF acid and FFCA generated also resulted in the lowering of pH in the media. To prevent these inhibitory effects, fed-batch experiments have been performed. The main problems overcome by pulse feeding of HMF and adjusting the pH with NaOH or HCl in the media. In fed-batch experiments, 30.1 g/L of FDCA is produced from HMF using glycerol as a carbon substrate at a yield of 97%. FDCA has been recovered from its culture broth as a pure dry powder (99.4%) using acid precipitation method followed by subsequent tetrahydrofuran extraction [65]. The 30.1 g/L FDCA yield is also suitable for laboratory level work which does not achieve the industrial need.

The genes encoding enzymes in the conversion of HMF into FDCA is identified in *Pencillium brasilianum* C1. Actual genes involved in HMF metabolism are hmfK1, hmfP, hmfK2, hmfL3, hmf14, hmfN2, hmfQ and hmfU. Besides, a few transport or regulation genes hmfR, hmfT3, hmfT4 and hmfT5 are also identified. The gene hmfK1 has a similar function to salicylate FDCA hydroxylase FAD decarboxylation binding monooxygenase which is involved in FDCA decarboxylation in the fungus. *C. basilensis HmfH* and HMF or FFCA aldehyde dehydrogenase gene is genetically expressed using expression construct comprises a G418 resistance marker and URA3 homologous site for chromosomal integration into the *S. cerevisiae* CEN.PK clone 2. *HmfH* gene construct consists of TEF1 promoter and by the CYC1 terminator for the transcription termination. HMF/FFCA aldehyde dehydrogenase gene is expressed from the TDH3 promoter and transcription is terminated by the TDH3 terminator. FDCA batch production of CEN.PK clone 2 is done in the mineral medium supplied with 1 g/L of glucose and 4 mM of HMF at 150 rpm yielded 0.22 mM FDCA after 40 h [66] and the yield is less as compared to other engineered microbial works.

Genes like hmfL1, hmfL2 and/or hmfN1 of *P. brasilianum* are expressed in *S. cerevisiae* CEN.PK. Resultant recombinant organisms are called as CEN.PK/PTT2, CEN.PK/PTT2-hmfN1-hmfL1-hmfL2, CEN.PK/PTT2-hmfN1-hmfL1 and CEN.PK/PTT2–hmfL1 respectively. Their activity has been checked in glucose-containing mineral medium with 3 mM HMF. Among them, the highest FDCA production (0.93 mM) is noticed by CEN.PK/PTT2-hmfN1-hmfL1. This FDCA production is increased up to 3.02 mM from 4 mM HMF after 40 hr by giving sufficient oxygen in the medium. *P. brasilianum* hmfL1 is also heterologously expressed in the yeast *Yarrowia lipolytica* Polg using pYLEX1-hmfL1 vector and conferred the ability to produce FDCA from HMF. This transformant was checked for activity in glucose-containing mineral medium supplemented with 4 mM HMF. It has shown 1.55 mM FDCA after 40 h upon HPLC analysis [66]. The work is much related to negligible FDCA yield with proof of concept.

HMF oxidase (HMFO) from *Methylovorus* sp. MP68 and HMF oxidoreductase (HmfH) from *Cupriavidus basilensis* HMF14 was inserted into *Raoultella ornithinolytica* BF60 by vector pBBR1MCS2 for improving FDCA production. After removal of *the LacZα* gene, the genes HmfH and HMFO are substituted and amplified. The locus *LacZα* is substituted with the genes HmfH or HMFO through recombination. Vector pMD19-T is used for the co-expression of these genes. The restriction-enzyme-ligation method is used for the preparation of this fusion fragment using *Hind* III, *Xba* I, and *Eco* RI restriction sites. The fusion fragment produced by recombination is inserted into the vector pBBR1MCS2 vector by using the recombination method and transformed into *Raoultella ornithinolytica* BF60. This engineered whole-cell biocatalyst has shown FDCA production from 51 mM (wild type strain) to 93.6 mM (recombinant strain). The experiments performed using pre-grown whole-cell biocatalyst and HMF in 50 mM phosphate buffer (pH 8.0) at 30°C showed a low level of intermediates like HMF alcohol and HMF [67]. This engineered strain produces a better amount of FDCA as compared to others with fast conversion.

#### 5.2.3. Engineering of genes into the metabolic pathways of strains for the production of FDCA

HMF is getting converted into FDCA metabolically, and after that, this FDCA may be up taken by the cells for its activities through TCA cycle. This is mainly done by the enzyme dicarboxylic acid decarboxylase (*dcaD*). Mutation of this gene has significantly increased the amount of FDCA in the medium. It has been mutated to block FDCA degradation to furoic acid in *R. ornithinolytica* BF60. Mutation of genes is usually done by mobile group II intron based genetic engineering systems. Gene targeting is mainly done by base pairing between the intron RNA and the targeted chromosomal DNA and thus the intron fragment can be modified easily [68]. Consecutive mutations with high efficiency could be done by Intron insertion into the chromosome and so it is a promising approach to marker less mutagenesis [69,70]. The intron sequences are amplified and digested with *Bsr* G1 and *Hind* III, and ligated with the pACD4K-C-loXp TargeTron plasmid and transformed into *E. coli* JM109 cells. These plasmids are transfected into *R. ornithinolytica* BF60 by electroporation. Intron-specific primers are used for the confirmation of successful insertion by using colony PCR. It is selected using Kanamycin selectable marker by Cre-loxP-mediated recombination. After *dcaD* mutation biotransformation of 100 mM HMF into FDCA is done in 50 mM phosphate buffer (pH 8) using 45 g/L recombinant *R. ornithinolytica* BF60 at 30°C. FDCA production is increased to 9.2 g/L. One side pathway is coming in HMF to FDCA biotransformation pathway, which reduces the production of desired product FDCA. So the HMF may biotransform into HMF alcohol instead of the desired pathway to FDCA through HMF acid or DFF. HMF to HMF alcohols is formed with the help of genes of alcohol reductase (*aldR*) in bacteria. So if these genes are mutated, FDCA production may also be increased. So the second targetron alcohol reductase (*aldR*) gene is also mutated [71] in *dca* mutated *R. ornithinolytica* BF60. These mutations are confirmed by probe labeling and southern blot analysis. This double mutant recombinant strain is used for the FDCA production analysis and the FDCA production is increased from 9.2 g/L to 11.3 g/L [61] which is not up to the level of industrial standards.

In the pathway of HMF metabolism into FDCA in *R. ornithinolytica* aldehyde dehydrogenase (*aldh1*) is responsible for the conversion FFCA to FDCA. So the overexpression of *aldh1* may be increased the FDCA production. Three aldehyde dehydrogenase genes (*aldhs*) are overexpressed using the vector pBBR1MCS-2 for *R. ornithinolytica* BF60. T7 lac or promoters are added into pBBR1MCS2 with *Sal* I restriction site. T7 RNA polymerase expressed pAR1219 helper plasmid transfected into the pBBR1MCS2 vector under the control of the IPTG-inducible lac UV51. The Amplified gene fragments are purified and ligated to a pMD19 simple T vector which is transfected into *E. coli*. These genes are re-amplified with restriction enzyme digestion sites using primers. It is ligated to pET 28a and pBBR1MCS-2 vectors after digestion and transfected into double mutant *R. ornithinolytica* BF60 (RTFB60-2). These *aldh1* recombinants are confirmed by restriction analysis and DNA sequencing. Expression of these three genes from *R. ornithinolytica* and enzymatic activities have been done by aldehyde reductase, decarboxylase and aldehyde dehydrogenase activity assays. After overexpression of *aldh1* into *R. ornithinolytica* BF60 (RTFB60-2) FDCA titer increased to 13.9 g/L which is almost 1.7 times greater than wild-type strain with the molar conversion of 89% from 51%[61].When the higher concentration of HMF (>100 mM) is used for the conversion of HMF into FDCA an inhibition is observed without its complete oxidation.

Fourteen gene expression cassettes are constructed to improve the FDCA production in *R. ornithinolytica* BF60. Genes, *hmfH* (HMF oxidoreductase) and *hmfo* (HMF oxidase) expression cassettes are constructed for fine-tuning of FDCA synthesis from HMF. The genes HMFO from *Methylovorus* sp. MP688 and HmfH from *Cupriavidus basilensis* HMF14 are synthesized and codon-optimized. It is expressed in *R. ornithinolytica* BF60. Plasmids pRSF, pACYC and pCDF, and are formulated after replacement of T7 promoter of the respected plasmids with promoters trc and/or tac based on pRSFDuet-1, pACYCDuet-1 and pCDFDuet-1 skeleton. They are electroporated into *R. ornithinolytica* BF60 and λ Red recombination system is used for gene deletion [72]. The recombinant strains are harvested after 24 h of incubation (OD_600_ of 100) and suspended (10 mL) in 50 mM phosphate buffer (pH 8.0) in 100 mL conical flasks. This strain improved the FDCA production of 108.9 mM with a yield of 73% from 150 mM HMF. It is almost a 16% higher yield than the non-recombinant strains. Ribosomal binding site sequences of *hmfH* are computationally designed using the RBS calculator and these sequences are assembled into *HmfH* expression cassettes. Ribosomal binding site sequences are inserted into plasmids by using a whole-plasmid PCR with suitable primers for the mutation sites. This expression cassette in *R. ornithinolytica* BF60 improved FDCA yield (93%) of 139.6 mM. Based on RNA-sequencing-based transcriptomics, it has been confirmed that genes dkgA, aldR, akR, adhP1, and adhP2 are responsible for the reduction of HMF into HMF alcohol in *R. ornithinolytica* BF60. These five genes are deleted by a method combinatorial deletion which led to less production of HMF alcohol from HMF. This led to enhancement (12%) in FDCA production of 175.6 mM. This FDCA synthesis is again improved by-fed batch strategy (50, 25, 25, 25, and 25 mM HMF in every 12 h from 24–72 h) and 221.5 mM FDCA produced with 88.6% yield from 250 mM HMF [73]. Even though the FDCA amount is approximate to 34.5 g/L (221.5 mM) its conversion rate (HMF to FDCA) is less as compared to other metabolically engineered strains.

## 6. Bioprocesses using recombinant and wild enzymes for the synthesis of FDCA

HMF oxidase is FAD-dependent oxidase from the family of the glucose-methanol-choline oxidoreductase, which has an oxidase activity on HMF to produce FDCA with molecular oxygen as a cofactor. HMF oxidase gene from *C. basilensis* HMF 14 is expressed in *E. coli* BL21. Affinity chromatography was used for the purification of this expressed gene. This expressed oxidase has the potential of oxidizing HMF alcohol to FDCA by four consecutive oxidations. Each oxidation step involves two-electron oxidation in which one molecule of O_2_ is utilized and one molecule of H_2_O_2_ is liberated by the enzyme. The highest yield (95%) of FDCA is obtained in reaction with 20 µM HMF oxidase and 20 µM FAD from 4 mM HMF after 15 h in 100 mM phosphate buffer (pH 7.0). The reaction is carried at ambient pressure (0.1 MPa) and temperature (25°C) and above 95% of yield is obtained if the reaction is kept for more than 24 h. HMF oxidase has wide substrate specificity for its sequential oxidations and product formation. Normally a proton is transferred from the alcohol group of any active site base followed by a hydride transfer (from ‘C’ atom of alcohol) to the FAD cofactor by FAD-dependent oxidases after alcohol oxidation [74]. Since this transfer is not possible in the case of aldehydes, most FAD-oxidases are not capable of aldehyde oxidations [75]. It has been confirmed that the full oxidation of HMF (which has aldehyde and alcohol moiety) to FDCA by this HMF oxidase. Even it has a higher range of substrate specificity, and aldehyde and alcohol oxidation of substrates at ambient temperature and pressure, it needs a longer reaction time of more than 24 h for the 100% product yield [76].

Three fungal aryl alcohol oxidase enzymes (*Pery*AAO, *Post*AAO and *Badu*AAO), recombinant galactose oxidase (expressed in *Aspergillus oryzae*) and peroxygenase from *Agrocybe aegerita* (AaeUPO) are tested for their capability to oxidize HMF into FDCA by multi enzyme cascade reaction processes. Three fungal aryl alcohol oxidase (AAO) enzymes *Pery*AAO, *Post*AAO and *Badu*AAO are produced and purified from wild fungi *Pleurotus eryngii, Pleurotus ostreatus* and *Bjerkandera adusta* respectively. Aryl alcohol oxidase enzymes are purified by ion-exchange chromatography steps using Q-sepharose and Mono Q columns followed a size exclusion chromatography using Sephadex 75 column. All these enzymes are analyzed for its conversion efficiency into suitable products for selecting the cascade system for the conversion to HMF to FDCA. Among the three fungal enzymes, *Pery*AAO which efficiently converts HMF to DFF is selected for the multi-enzyme cascade reactions. AaeUPO is selected because of its efficiency in the conversion of FFCA to FDCA which also utilizes the H_2_O_2_ produced by the oxidase in the sequential oxidation steps. Galactose oxidase (belongs to the copper radical oxidases family) is selected due to its capacity to oxidize HMF acid to FFCA. So after all the preliminary analysis of these three enzymes, a multi-enzyme reaction set up is converted 9.7 mM HMF into 7.9 mM of FDCA after 24 h with 80% yield in 50 mM phosphate buffer (pH 7.25) [77]. Here, recombination and purification of three enzymes for the conversion of HMF into FDCA will not be economically viable for the large scale processes. Also, 80% conversion of HMF into desired product FDCA happened after 24 h which made this into a slow process.

Oxidative conversion of HMF into FDCA is taken place by an enzymatic cascade system using the enzymes Aryl alcohol oxidase (AAO) from *P. eryngii* and Peroxygenase (UPO) from *Agrocybe aegerita*. AAO can transform HMF and DFF by oxidation in the presence of O_2_ and, FFCA and H_2_O_2_ are formed. HMF to FFCA conversion is taken place after four successive electron oxidations by AAO. Here UPO mainly catalyzes the conversion of HMF into HMF acid and FFCA to FDCA with the expense of H_2_O_2_, produced from the AAO reactions. After enzyme cascade reaction simultaneous action of AAO and UPO on HMF have not improved FDCA yield. It might be because of UPO oxidization of HMF to HMFCA took place with the expense of H_2_O_2_, which is produced by the AAO after HMF to FFCA conversion. So there may be a limited amount of H_2_O_2_ for the action of UPO for the conversion of FFCA to FDCA. In the enzyme cascade system using *P. eryngii* AAO (5 µM) and *A. aegerita* UPO(0.65 µM), 91% FDCA is obtained from 3 mM HMF after 120 h in 5 mL phosphate buffer (pH 6.0–7.0) at 25°C [78]. It is a long term reaction to the production of FDCA with low substrate concentration.

The enzymatic cascade is involved with three fungal oxidoreductases for the production of FDCA from a new substrate 5-methoxymethylfurfural (MMF). MMF is converted into MMFA (5-methoxymethylfurancarboxylic acid) followed by HMF acid, FFCA and FDCA after sequential oxidations. Three fungal enzymes are involved in this cascade system Aryl-alcohol oxidase (AAO), unspecific peroxygenase (UPO) and Methanol oxidase (MOX). The gene (CDNA) coded for AAO from *P. eryngii* is expressed in *E. coli* W3110 harboring the pFLAG1 vector. *A. aegerita* UPO gene is inserted into the vector pPICZ-B-PaDa-I and heterologously expressed in *P. pastoris*. The enzyme is purified using Sepharose FF and Q-source chromatography columns. MOX is available commercially made from *Pichia pastoris*. MOX is taken methanol released for *in situ* producing H_2_O_2_ which is produced by aryl-alcohol oxidase acts as fuel for the peroxygenase reactions. Conversion of MMF into FDCA is taken place after three or four sequential oxidation steps. It mainly depends upon whether the breakdown of ether leaves alcohol or a carbonyl function in the furfuraldehyde. Enzyme cascade reaction of AAO (5 μM), UPO (5 μM) along with MOX (1 μM) have converted 1.5 mM MMF into 98% FDCA after 120 h of incubation. This reaction is carried out in 100 mM phosphate buffer (pH 7.0) in the presence of H_2_O_2_ (1 mM) and methanol (1 mM) at temperature 28°C [79]. This reaction takes place with the action of expensive three enzymes, low substrate concentration and long reaction time. Also, the substrate MMF has not much advantage than the commonly used substrate HMF for the production of FDCA.

An enzyme cascade reaction using this periplasmic aldehyde oxidase, galactose oxidase M3–5 and catalase is performed for the conversion of HMF into FDCA. It has been reported that *E. coli* periplasmic aldehyde oxidase (PaoABC) is a 135 kDa heterotrimeric enzyme that uses oxygen as the electron acceptor has the capability to convert toxic aldehydes into nontoxic intermediates. It has a large molybdenum cofactor containing PaoC subunit (78.1 kDa), a medium-sized FAD containing PaoB subunit (33.9 kDa), and a small [2Fe–2S] PaoA subunit (21.0 kDa) [80,81]. His tag fused (N terminal) PaoABC genes heterologously expressed into *E. coli* and transformed into *E. coli* TP1000 cells (deleted mobAB genes). This His-tagged PaoABC is eluted in column chromatography with 100 mM imidazole in 50 mM sodium phosphate (pH 8.0) and 300 mM NaCl. Galactose oxidase variant M3-5 gene (GOase M3-5) is transformed into *E. coli* BL21 according to manufactures protocol (Invitrogen). The enzyme is purified with a 5-mL-Strep-Tag-II column (GE Healthcare) pre-equilibrated with buffer 50 mM sodium phosphate and 300 mM NaCl. It is followed by dialysis in 30 kDa cutoff dialysis membrane and eluted. Along with these, purified PaoABC, GOase M3-5 and catalase (Sigma) are also used for the experiments. In a one-pot sequential reaction GOase M3-5 (3.3 mg/mL), catalase (3.3 mg/mL) and 50 mM HMF are added into 400 mM potassium phosphate buffer (pH 7.0) and incubated at 37°C in a shaking incubator. After all HMF is oxidized, PaoABC (13.3 mg/mL) is added into this reaction mixture and placed back into the shaking incubator for the analysis of intermediates. After 8 h 90% FDCA is produced with 100% HMF conversion [82]. The use of three enzymes and low substrate concentration will not make the process economically viable. But, multiple substrate specificities of PaoABC oxidation led to the conversion of toxic substrates into desired products that take advantage in the future.

Tandem oxidations of Galactose oxidase (GO) and lipases are designed for the conversion of HMF to FDCA. GO, (8 U) is incubated in deionized water and sodium acetate buffer (50 mM) to replace phosphate buffer. The DFF yield (92%) is increased after 96 h from 30 mM HMF. After this preliminary analysis for the tandem oxidations HMF to FDCA, HMF is transformed into DFF by GO after 48 h with 75% conversion efficiency. This DFF (22.5 mM) is oxidized using Immobilized lipase B (9.6 mg) from *Candida antartica* (CAL-B, Novozyme) and H_2_O_2_ (30% v/v) and complete uptake of DFF is taken place within 7 h in 2 mL butanol-Ethanol (1:1, v/v). This DFF is converted into FFCA as an intermediate which is completely transformed into 88% of FDCA after 24 h at 40°C [83].

Four enzymes, periplasmic aldehyde oxidase (PaoABC), galactose oxidase M3-5, horseradish peroxidase and catalase are used for the biotransformation of HMF into FDCA as Continuous one-pot reaction set up. It is known that Horseradish peroxidase (HRP) can activate GOase, was used in this reaction. Here PaoABC uses atmospheric oxygen from the air as an electron acceptor. The reaction was carried out in 200 mM phosphate buffer (pH 7.0) using enzymes PaoABC (28.9 mg/mL), GOase M3-5 (3 mg/mL), catalase (3.30 mg/mL) and HRP (1 mg/mL) at 37°C. This enzyme cascade system has converted 50 mM HMF into FDCA (100% yield) after 6 h [84]. It is noted that the unwanted formation of HMF acid during FDCA synthesis is avoided by the stepwise addition of the enzymes. The use of atmospheric oxygen has resulted in a higher rate of HMF transformation and therefore higher substrate concentration is also used in this study (> 100 mM HMF).

Combined-cross linked enzyme aggregates (combi-CLEAS) are multi-functional biocatalyst used for the formulation of immobilization of two or more enzymes. Also, combi-CLEAS are applied as versatile carrier-free immobilized systems. So these can be used for merging multi-step enzyme cascade and non-enzyme cascade biotransformations of HMF into one pot reaction [85,86]. PaoABC is immobilized into PaoABC-Gel and catalase is entrapped into CLEA and both CAT-CLEA and PaoABC-Gel are joined to form a complex. This PaoABC-Gel/CAT-CLEA complex is capable of tolerating higher substrate concentration (DFF) up to 200 mM with a recyclable capacity of 14 times without loss in product (FDCA) yield [84].

## 7. Challenges on the green synthesis of FDCA

The reports suggest that the green synthesis of FDCA is possible by using microorganisms (Table 2) and enzymes (Table 3). Still, none of the industries have established a lab for FDCA by complete green technologies. So far these industries are trying to produce FDCA from lignocellulosic biomass waste and cheap sources by partial chemical treatments with mild or toxic chemicals. Apart from that biological synthesis of FDCA favors nontoxic media ingredients. In the case of microbial assisted FDCA production, the reported microorganisms’ show improved yield after media optimizations, genetic engineering and metabolic engineering approaches. But the highest production of FDCA by these microorganisms happens after 2–3 days, which is a drawback for the large scale production. Hence, the microbial conversion of HMF into FDCA is a time-consuming process. Even the conversion of HMF to FDCA is complex biotransformation, intermediates like DFF and FFCA get transformed into FDCA quickly as compared to HMF acid which interferes or less convert into FDCA. HMF acid is seen in the extracellular medium of both bacteria and fungus along with HMF alcohol, FFCA and FDCA during the biotransformation process. Uptake of HMF is faster but the extracellular HMF acid uptake is a slower process for all microorganisms which is a rate-limiting step and time consuming for the biotransformation of HMF into FDCA. It might be due to the less toxicity of HMF acid to bacteria or fungi and the non-availability of enzymes for its alcohol group to aldehyde group conversion. If the absence of carbon source in the medium, the produced FDCA might be taken up by microorganisms and will be progressing through the TCA cycle for is metabolism. So media engineering plays an important role with microbial growth conditions along with HMF biotransformation focusing on FDCA synthesis.

**Table 2.** Microbial technology for the biotransformation of HMF into FDCA.

Sl. No	Name of the organisms	Media	HMF (g/L)	FDCA (g/L)	Yield (%)	Time (h)	Reference
1	*B. cepacia* H 2	MSM	2	1.27	51.21	24	[59]
2	*B. cepacia* H 2	Algal acid hydrolyzate	2	1.03	41.53	18	[59]
3	*M. radiotolerans* G-2	MSM	3	1.6	43.01	72	[60]
4	*M. radiotolerans* G-2	Algal acid hydrolyzate	3	0.45	12.1	-	[60]
5	*R. ornitholytica* BF 60	Phosphate buffer	12.6	9.2	58.88	14	[61]
6	*Enterobacter* sps	MSM	0.5	0.07	11.29	336	[62]
7	*A. oleiovirans* S 27	MSM	0.5	0.4	64.52	24	[63]
8	*A .flavus* APLS-1	MSM	1	0.83	66.93	336	[58]
9	*R .ornitholytica* BF 60	Sodium phosphate buffer	35	41.29	95.14	72	[64]
10	*P. putida* S 12	Glycerol + HMF	23	30.1	97	144	[65]
11	*P. brasilanam* CEN.PK Clone 2	Glucose + HMF	0.5	0.03	4.84	40	[66]
12	*P. brasilanam* CEN.PK/PTT2-hmfN1-hmfL1	Glucose + HMF	0.5	0.47	75.81	40	[66]
13	*Y. lipolytica* pol g PVLEX1-hmfL1	Glucose + HMF	0.5	0.24	38.71	40	[66]
14	*R. ornitholytica* BF 60	Phosphate buffer	12.6	14.6	93.45	144	[67]
15	*R. ornitholytica* BF 60	Phosphate buffer	12.6	11.3	72.32	144	[61]
16	*R. ornitholytica* BF 60	Phosphate buffer	12.6	13.9	88.96	144	[61]
17	*R. ornitholytica* BF 60	Phosphate buffer	32	34.5	88.6	144	[73]

**Table 3.** Enzymatic technologies for the biotransformation of HMF into FDCA.

Sl. No	Name of the enzymes	Media	HMF (g/L)	FDCA (g/L)	Yield (%)	Time (h)	Reference
1	HMF oxidase	Phosphate buffer	0.5	0.59	95	15	[76]
2	1) Pery AAO	Phosphate buffer	1.2	1.21	80	24	[77]
	2) Aae UPO						
	3) Galactose oxidase						
3	1) *P. eryngii* AAO						
	2) *A. aegerita* UPO	Phosphate buffer	0.39	0.43	91	120	[78]
4	1) Aryl alcohol oxidase						
	2) Unspecific peroxyganse	Phosphate buffer	0.19	0.23	98	120	[79]
	3) Methanol oxidase						
5	1) Periplasmic aldehyde oxidase (PaoABC)						
	2) Galactose oxidase M3-5						
	3) Catalase	Phosphate buffer	6.3	7	90	8	[82]
6	1) Galactose oxidase	Butanol-Ethanol & Sodium acetate buffer					
	2) Lipase		3.78	4.12	88	48	[83]
7	1) PaoABC						
	2) Galactose oxidase M3-5	Phosphate buffer	6.3	7.81	100	6	[84]
	3) Horseradish peroxidase						
	4) Catalase						

Strategies like media engineering, genetic engineering, and metabolic engineering have made HMF tolerant microorganisms with higher FDCA yield. Reports are concentrating on batch and fed-batch processes only, which have limitations. Still, this substrate tolerant concentration and FDCA production are not enough for the large scale production of industries. Another concern is that most of the microbial biotransformation works have been carried out in the flask level only. These works have not yet attempted in fermenters. Pilot scale production of FDCA is yet to be established. And the techno-economic analysis (TEA) and Life cycle assessment [76] of FDCA production through microbial transformations are also needed to be carried out with its environmental effects. This will provide clues of economic feasibility of the process and fine tuning can be done at unit operations level for improving the overall process economics.

Enzyme biotransformations are fast and not time-consuming. But most of the works have been successively carried out with recombinant and wild enzymes through one pot or enzyme cascade system which makes enzyme assisted biotransformations as non-economic. Also, inhibitors like H_2_O_2_ hinder its activity and reduce substrate binding and product formation. So this should be eliminated or used by another enzyme using H_2_O_2_. Alcohol to aldehyde and aldehyde to acid group transformation of HMF and its intermediates are performed using multiple enzymes, which make the sequential reaction as a complex one. Enzymes have higher substrate tolerance and high yield but all the reactions are in laboratory-scale only. Also, these experiments or reactions are carried out in milliliters only. So this will be another challenge for the biotransformation of HMF into FDCA using enzymes from the infant stage to pilot scale with TEA and LCA analysis. Addressing these challenges will make the process economically viable.

## 8. Conclusion

Biotransformations of HMF into FDCA have been done by microorganisms and enzymes so far. Normal microbial HMF biotransformations and FDCA production rates are enhanced by engineering advancements like media engineering, genetic engineering and metabolic engineering. The use of recombinant and wild enzymes has led into the development of new enzyme cascade systems for higher substrate tolerance and product yield with less time. Since it is economically feasible with better yield, most of the industries still depend on chemical processes. Globally, there will be a need of 4 metric tons of FDCA per year but have not covered its one quarter yet by these chemical and biological processes. Though several research activities are going on throughout the world for FDCA production, still the green strategy for FDCA production is not economic and not up to the mark of industrial market level. It may take a long way from laboratory to industrial scale, but the use of toxic chemical catalysts give the way for ‘greener technologies’ in the coming years without any hazardous effects for the natural environment. If the challenges are overcome by recent technologies with some improvements in microbial and enzyme processes, it might result in higher FDCA yield and reach up to the level of the global market in the near future.

## 9. Future prospective

So far none of these green technologies have been taken up by industries due to fewer yields. But this routine production can be enhanced again with new potent isolated strains, finding pathways for the FDCA production in new strains followed by media, genetic and metabolic engineering. Also, the microbial production can be enhanced after protein engineering of transport proteins for HMF, HMF alcohol, HMF acid, DFF, FFCA, and FDCA. This protein engineering of transport proteins can uptake more substrates within a limited time. This may change the site of microbial FDCA production as periplasmic, intracellular or extracellular completely rather than three sites together for the conversion of HMF into FDCA. Also, FDCA consumption to 2-furoic acid and HMF to HMF alcohol conversion can be arrested and the yield will be increased in the medium by this protein engineering of transport proteins in the future. Also, HMF toxicity can be overcome by fed-batch or continuous fermentation modes for large scale FDCA production.

The fast-acting greener enzymes on HMF to FDCA production will be more advantageous if the enzymes are reusable. The immobilization of the enzymes on the desired substratum without loss of its activity will be one of the good techniques to recover from economic loss for the industries. Also, the development of multiple substrate specificity enzymes will be more economic for the industries than single substrate specificity which can produce different types of industrially relevant products. Another one of the future aspects of FDCA production using enzymes are enzyme engineering for different substrates in the pathway. Here the HMF conversion to FDCA pathway intermediates has alcohol and aldehyde, functional group. One engineered enzyme which is capable of higher binding affinity with all these intermediates and converts HMF into FDCA through its intermediates can tackle this issue in the future. The active site of this engineered enzyme can bind to all intermediates immediately and convert alcohol or aldehyde group into the desired aldehyde and acid group. So the use of multiple enzyme cascade systems with three or four enzymes for the conversion of HMF into FDCA can be minimized with the use of one enzyme. Tackling these bottlenecks will result in developing an eco-friendly and economically feasible strategy for the production of low volume high-value compounds shortly.

## Highlights

- Conversion of biomass into 2, 5-FDCA through partial chemical and greener approaches.
- Greener synthesis of 2, 5-FDCA from HMF through microbial biotransformation.
- Greener synthesis of 2, 5-FDCA from HMF through enzymatic biotransformation.
- Bioengineering approaches for enhanced production of 2, 5-FDCA.
